# Differences in pregnancy complications and outcomes by fetal gender among Japanese women: a multicenter cross-sectional study

**DOI:** 10.1038/s41598-020-75969-8

**Published:** 2020-11-02

**Authors:** Satoru Funaki, Kohei Ogawa, Nobuaki Ozawa, Aikou Okamoto, Naho Morisaki, Haruhiko Sago

**Affiliations:** 1 Center for Maternal-Fetal, Neonatal and Reproductive Medicine, National Center for Child Health and Development, 2-10-1 Okura, Setagaya-ku, Tokyo, 157-8535 Japan; 2 Department of Obstetrics and Gynecology, The Jikei University School of Medicine, 3-25-8 Nishi-Shimbashi, Minato-ku, Tokyo, 105-8461 Japan; 3 Department of Social Medicine, National Research Institute for Child Health and Development, 2-10-1 Okura, Setagaya-ku, Tokyo, 157-8535 Japan

**Keywords:** Medical research, Risk factors

## Abstract

The association between fetal gender and rare pregnancy complications has not been extensively investigated, and no studies have examined this association in Japanese women. Thus, we used a large Japanese birth registry database to investigate the extent to which fetal gender affects various pregnancy outcomes. We analyzed 1,098,268 women with a singleton delivery with no congenital anomaly at 22 weeks or later between 2007 and 2015. Women carrying a male fetus had a significantly higher risk of placental abruption (adjusted risk ratio [aRR] 1.15, 95% confidence interval (CI) 1.10–1.20)], preterm delivery (aRR 1.20, 95% CI 1.19–1.22), instrumental delivery (aRR 1.27, 95% CI 1.26–1.29), and cesarean delivery (aRR 1.01, 95% CI 1.00–1.02). In contrast, they had a significantly lower risk of preeclampsia (aRR 0.92, 95% CI 0.89–0.94), placenta accreta (aRR 0.90, 95% CI 0.85–0.96), atonic hemorrhage (aRR 0.95, 95% CI 0.93–0.96), and maternal blood transfusion (aRR 0.95, 95% CI 0.92–0.99). Our findings demonstrate a significant association between fetal gender and various pregnancy complications and delivery outcomes among Japanese women.

## Introduction

Some studies have investigated the association between fetal gender and pregnancy outcomes^1,2^. While these studies consistently showed that women carrying a male fetus were more likely to have an increased risk of preterm delivery^3–5^, premature rupture of membranes^6,7^, gestational diabetes mellitus (GDM)^8–10^, fetal macrosomia^6,9,10^, cord complications^9,10^, and instrumental and cesarean delivery^6,8–11^, studies of preeclampsia risk have shown inconsistent results^8–10,12^. Furthermore, the association between fetal gender and certain important outcomes, such as placenta accreta and abruption, has not been extensively studied, possibly due to their rarity.

Most studies to date have been primarily conducted in Western countries, although the association between fetal gender and pregnancy outcomes may differ by ethnicity^13^. Two studies enrolling Chinese subjects had non-negligible limitations, including a higher rate of cesarean section and distortion of the male:female ratio^14,15^. These are the only studies to date that have been conducted in Asia. Thus, replicative studies on an Asian population are required using other databases with minimum bias.

Our study used a large Japanese birth registry database to examine the extent to which fetal gender affects outcomes.

## Methods

### Study population

We used data from the Japan Society of Obstetrics and Gynecology (JSOG) perinatal database, which has been described elsewhere in detail^16^. Briefly, the database is an ongoing registry that began in 2001 and is based on the collaboration of 149 tertiary hospitals (as of 2012), covering nearly 15% of the annual births in Japan. The database contains transcribed data, including maternal demographics, pregnancy complications, and delivery outcomes extracted from the medical records at each hospital. The registry of the JSOG perinatal database was conducted in accordance with the guidelines of the Declaration of Helsinki and other nationally valid regulations. Informed consent was obtained in the form of an opt-out on the web site.

Our sample population was restricted to women who delivered singletons with no congenital anomaly between 2007 and 2015, because the effect of fetal gender may differ in cases of multiple pregnancies and fetal malformations. Women with missing data on fetal gender and gestational age at birth as well as women with unreliable data on the combination of birth weight and gestational age were excluded according to criteria proposed by a previous study^17^. Our main analysis of pregnancy complications was based on the women remaining after excluding those with missing or unreliable data on maternal age, parity, maternal height, pre-pregnancy body mass index (BMI), conception method, delivery mode, and fetal presentation at birth. We considered “missing” as an independent status for maternal smoking during pregnancy as these data were missing in a large number of women. Women who experienced intrauterine fetal death were also excluded from our analysis of delivery outcomes. Our analysis of instrumental deliveries excluded those with a cesarean section.

We conducted a sensitivity analysis to assess for any possible bias due to missing data based on all women regardless of missing or unreliable data. Missing or unreliable data were replaced with imputations for maternal age, parity, maternal height, pre-pregnancy BMI, conception method, delivery mode, and fetal presentation at birth. We created 30 sets of imputed datasets using multivariate imputation by chained equations.

### Variables of interest

The primary outcomes of interest were pregnancy complications and delivery outcomes. Preeclampsia, hemolysis, elevated liver enzymes, and low platelet count (HELLP) syndrome, placenta previa, placenta accreta, placental abruption, fetal death in utero, chorioamnionitis (CAM), and amniotic fluid embolism were defined as pregnancy complications, whereas preterm birth, fetal macrosomia, fetal presentation at birth, hypotonic and hypertonic uterine inertia, atonic hemorrhage, blood transfusion at birth, and instrumental or cesarean delivery were defined as delivery outcomes. Unfortunately, the information of GDM was not included in our database. Thus, we were unable to include GDM as a pregnancy complication of interest. Preterm delivery, very preterm delivery, and extremely preterm delivery were defined as less than 37, 32, and 28 completed weeks of gestation, respectively. Macrosomia and low birth weight were defined as a birth weight ≥ 4000 g and < 2500 g^18^, respectively. Preeclampsia was diagnosed when a systolic/diastolic blood pressure > 140/90 mmHg emerged after 20 weeks’ gestation with significant proteinuria (≥ 300 mg/day) in accordance with the national guidelines^19^. Similarly, obstetricians clinically diagnosed HELLP syndrome and CAM according to the criteria in the national guidelines. HELLP syndrome was diagnosed by a combination of elevated liver enzymes (both aspartate transaminase > 70 IU/L and lactate dehydrogenase > 600 IU/L), thrombocytopenia (< 100,000/µL), and decreased anti-thrombin III activity (< 60%)^20^. CAM was diagnosed by the presence of maternal fever with one or more of the following symptoms: maternal tachycardia (≥ 100 times/min), uterine tenderness, maternal white blood cell count > 15,000/mm^3^, and purulent discharge^19^. Obstetricians at each hospital reported the other variables by checking against the items in the JSOG database based on their clinical diagnosis.

Among the other covariates, parity was classified as either “0” or “1 or above.” The conception method was categorized as “with assisted reproductive technology (ART)” or “without ART,” and smoking status during pregnancy was categorized as “yes,” “no,” or “missing.”

### Statistical analysis

First, the maternal demographics and birth year by fetal gender were compared using Student’s *t*-test for continuous variables and the chi-square test for categorical variables. We used linear regression analysis to calculate the mean difference for continuous variables. Second, we used multivariate Poisson regression analysis for categorical variables and linear regression analysis for continuous variables to estimate the association between fetal gender and pregnancy complications as well as delivery outcomes using the female fetus as a reference. Maternal age, maternal height, pre-pregnancy BMI, parity, conception method, and smoking status during pregnancy were considered potential confounding factors in the multivariate analysis. Although some studies have shown that sex selection may be influenced by environmental factors, such as the conception method^21^ and maternal periconceptional smoking status^22^, there is a lack of studies on significant associations between maternal factors (such as maternal age, height, and BMI) and fetal sex. However, we considered that the adjustment of these factors was important as there is insufficient evidence showing that they are independent of fetal sex. To confirm the results of our analyses after excluding women with missing or unreliable data, we conducted a sensitivity analysis of the same cohort and imputed the missing or unreliable data.

All descriptive and statistical analyses were performed using STATA v15 (STATA Corp, College Station, TX). Each result was presented as a risk ratio and 95% confidence interval (CI). A p-value < 0.05 was considered statistically significant.

### Study approval

The study protocol was approved by the Institutional Review Board of the National Center for Child Health and Development on November 29, 2018 (no. 1983).

## Results

**Figure 1:**
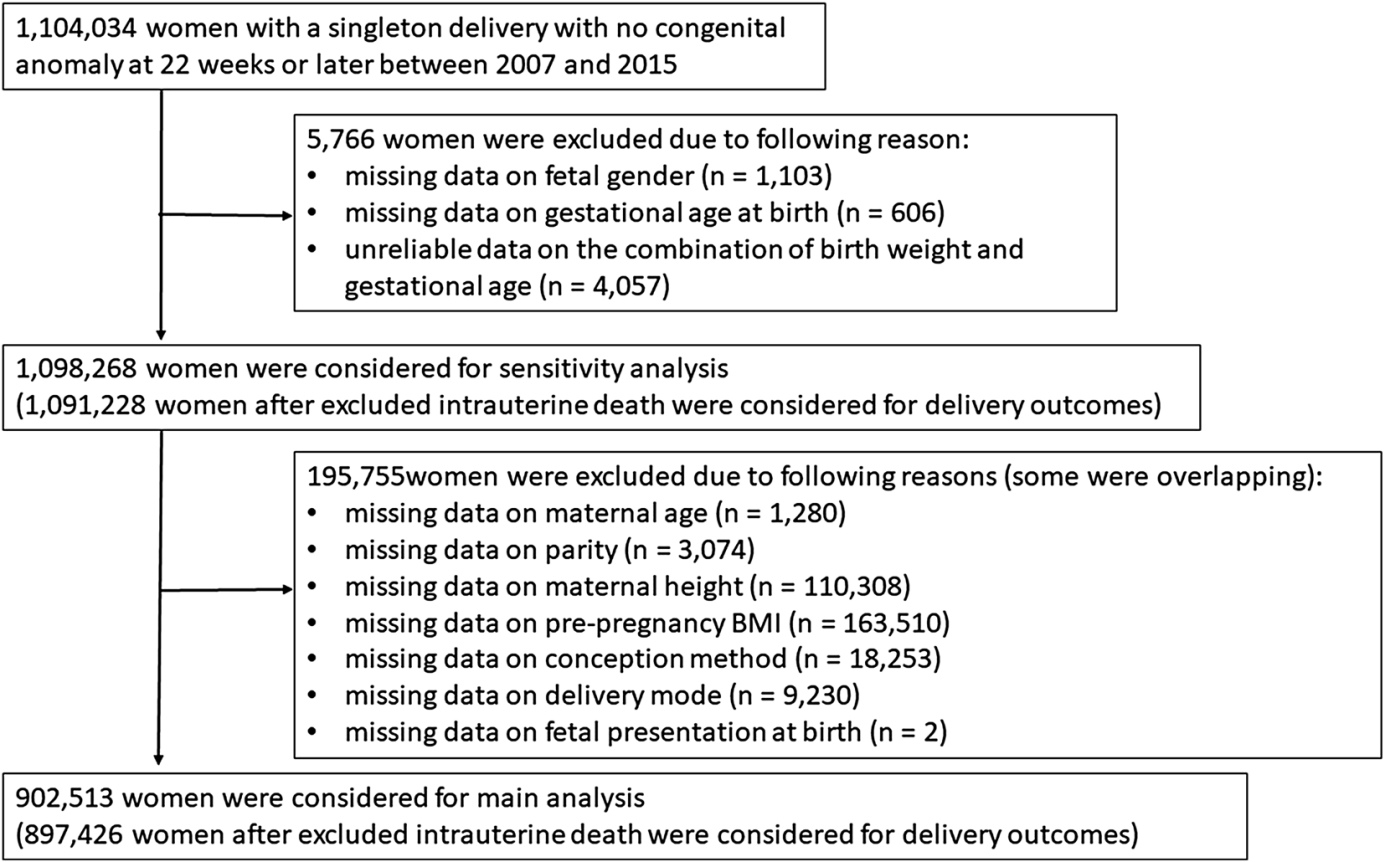
Flow chart showing the study population selection. The main analyses were conducted based on 902,513 women (897,426 were used for delivery outcomes, after excluding those with intrauterine death) after excluding those with missing data on fetal gender and gestational age at birth and unreliable data on the combination of birth weight and gestational age. Sensitivity analyses were conducted based on 1,098,268 women (1,091,228 for delivery outcomes) after excluding missing data on maternal age, parity, maternal height, pre-pregnancy BMI, conception method, delivery mode, and presentation at birth.

We analyzed 1,104,034 women with a singleton delivery with no congenital anomaly at 22 weeks or later between 2007 and 2015. We excluded 1103 (0.10%) with missing data on fetal gender, 606 (0.05%) with missing data on gestational age at birth, and 4057 (0.37%) with unreliable data on the combination of birth weight and gestational age. The main analysis was conducted on 902,513 of the remaining 1,098,268 women after excluding those with missing or unreliable data on maternal age, parity, maternal height, pre-pregnancy BMI, conception method, delivery mode, and fetal presentation at birth (Fig. 1).

There were 464,075 (51.4%) women carrying a male fetus and 438,438 (48.6%) carrying a female fetus. Table 1 shows the maternal characteristics stratified by fetal gender. Non-significant differences by fetal gender were observed in terms of maternal age, parity, conception method, maternal height, and smoking status. However, women carrying a male fetus had a significantly higher pre-pregnancy BMI (p = 0.005) with a mean difference of 0.02 kg/m^2^ (mean pre-pregnant BMI among women carrying a male fetus vs. female fetus was 21.24 kg/m^2^ vs. 21.22 kg/m^2^).

**Table1:** Demographics of pregnant women stratified by fetal sex. (n = 902,513).

Variables	Fetal sex		Mean difference mean (95% CI)	p-value^a^
	Male (%) (n = 464,075)	Female (%) (n = 438,438)		
**Continuous variables [Mean (n)]**				
Maternal age (years)	32.0 (5.3)	32.1 (5.4)	0.00 (− 0.03 to 0.02)	0.165
Pre-pregnant body mass index (kg/m^2^)	21.24 (3.47)	21.22 (3.47)	0.02 (0.01 to 0.04)	0.005
Maternal height (cm)	158.3 (5.5)	158.3 (5.5)	− 0.01 (− 0.03 to 0.01)	0.394
**Categorical variables [n (%)]**				
Year				0.273
2007–2009	76,087 (51.6)	71,337 (48.4)		
2010–2012	136,261 (51.4)	128,856 (48.6)		
2013–2015	251,727 (51.4)	238,245 (48.6)		
Parity				0.066
Primiparous	240,523 (51.5)	226,388 (48.5)		
Multiparous	223,552 (51.3)	212,050 (48.7)		
Conception method				0.928
Without ART	445,831 (51.4)	421,218 (48.6)		
With ART	182,44 (51.4)	17,220 (48.6)		
Maternal smoking during pregnancy				0.488
Yes	21,223 (51.7)	19,833 (48.3)		
No	353,727 (51.4)	334,502 (48.6)		
Missing	89,125 (51.5)	84,103 (48.6)		

All mean differences are calculated using females as the reference.

*CI* confidence interval, *BMI* body mass index, *ART* assisted reproductive technology.

^a^Student's *t*-test was used for continuous variables and the chi-squared test for categorical variables.

Table 2 shows the association between fetal gender and pregnancy complications. Women carrying a male fetus had a significantly higher risk of placental abruption (0.85%, male and 0.74%, female; adjusted risk ratio [aRR] = 1.15, 95% CI 1.10–1.20). In contrast, women carrying a male fetus had a significantly lower risk of preeclampsia (2.00%, male and 2.17%, female; aRR 0.92, 95% CI 0.89–0.94) and placenta accreta (0.42%, male and 0.47%, female; aRR 0.90, 95% CI 0.85–0.96). We did not detect any significant association between fetal gender and other pregnancy complications, including HELLP syndrome, placenta previa, intrauterine fetal death, CAM, and amniotic fluid embolism.

**Table 2:** Association between fetal sex and pregnancy complications. (n = 902,513).

Outcomes	Male (%) (n = 464,075)	Female (%) (n = 438,438)	Crude RR (95% CI)	Adjusted RR^a^ (95% CI)
Preeclampsia	9265 (2.00)	9502 (2.17)	0.92 (0.89–0.95)	0.92 (0.89–0.94)
HELLP syndrome	565 (0.18)	509 (0.17)	1.05 (0.93–1.18)	1.05 (0.93–1.18)
Placental abruption	3946 (0.85)	3250 (0.74)	1.15 (1.10–1.20)	1.15 (1.10–1.20)
Placenta accreta	1941 (0.42)	2040 (0.47)	0.90 (0.84–0.96)	0.90 (0.85–0.96)
Placenta previa	7312 (1.58)	6876 (1.57)	1.00 (0.97–1.04)	1.01 (0.97–1.04)
Intrauterine fetal death	2641 (0.57)	2446 (0.56)	1.02 (0.97–1.08)	1.02 (0.96–1.08)
Chorioamnionitis	4187 (0.90)	3843 (0.88)	1.03 (0.99–1.08)	1.03 (0.98–1.07)
Cord prolapse	136 (0.04)	104 (0.04)	1.24 (0.96–1.60)	1.24 (0.96–1.60)
Amniotic fluid embolism	50 (0.01)	51 (0.01)	0.93 (0.63–1.37)	0.93 (0.63–1.37)

All risk ratios are calculated with females set as the reference.

*RR* risk ratio, *CI* confidence interval, *HELLP* hemolysis, elevated liver enzymes, low platelet count.

^a^Adjusted for maternal age, maternal height, maternal pre-pregnant body mass index, parity, conception method, and maternal smoking status during pregnancy.

Table 3 shows the association between fetal gender and delivery outcomes among women with live birth. The analyses on delivery outcomes were based on 897,426 women after excluding those with intrauterine fetal death. The analysis of instrumental deliveries was restricted to 632,659 women, excluding those with a cesarean section. Women carrying a male fetus had a higher risk of preterm delivery (12.27%, male and 10.20%, female; adjusted odds ratio (aOR): 1.20, 95% CI 1.19–1.22), very preterm delivery (2.37%, male and 2.13%, female; aOR 1.11, 95% CI 1.08–1.14), and extremely preterm delivery (0.77%, male and 0.72%, female; aOR 1.08, 95% CI 1.02–1.13). They also had a higher risk of fetal macrosomia (0.99%, male and 0.54%, female; aOR 1.83, 95% CI 1.73–1.91), hypotonic uterine inertia (9.50%, male and 8.89%, female; aOR 1.07, 95% CI 1.05–1.08), hypertonic uterine inertia (0.11%, male and 0.09%, female; aOR 1.21, 95% CI 1.06–1.37), instrumental delivery using vacuum and forceps (11.17%, male and 8.79%, female; aOR 1.27, 95% CI 1.26–1.29), and cesarean delivery (29.63%, male and 29.37%, female; aOR 1.01, 95% CI 1.00–1.02). However, women carrying a male fetus had a lower risk of low birth weight (14.55%, male and 16.87%, female; aOR 0.84, 95% CI 0.83–0.85), non-cephalic position at birth (5.36%, male and 6.16%, female; aOR 0.87, 95% CI 0.85–0.88), atonic hemorrhage (5.18%, male and 5.47%, female; aOR 0.95, 95% CI 0.93–0.96), and maternal blood transfusion (1.09%, male and 1.14%, female; aOR 0.95, 95% CI 0.92–0.99). The analysis of continuous variables showed that the gestational age at birth was lower (adjusted mean difference: − 0.18 weeks, 95% CI − 0.19 weeks to − 0.17 weeks) and the birth weight was higher (adjusted mean difference: 77.3 g, 95% CI 75.1–79.5 g) among women carrying a male fetus.

**Table 3:** Association between fetal sex and delivery outcomes among women with live birth. (n = 897,426).

Outcomes	Male (%) (n = 461,434)	Female (%) (n = 435,992)	Crude RR (95% CI)	Adjusted RR^a^ (95% CI)
**Categorized variables [n (%)]**				
Preterm delivery (< 37 weeks)	56,604 (12.27)	44,476 (10.20)	1.20 (1.19–1.22)	1.20 (1.19–1.22)
Very preterm delivery (< 32 weeks)	10,929 (2.37)	9279 (2.13)	1.11 (1.08–1.14)	1.11 (1.08–1.14)
Extremely preterm delivery (< 28 weeks)	3561 (0.77)	3126 (0.72)	1.08 (1.03–1.13)	1.08 (1.02–1.13)
Macrosomia	4575 (0.99)	2369 (0.54)	1.82 (1.74–1.92)	1.83 (1.73–1.91)
Low birth weight	67,151 (14.55)	73,561 (16.87)	0.84 (0.83–0.85)	0.84 (0.83–0.85)
Non-cephalic position at birth	24,715 (5.36)	26,868 (6.16)	0.87 (0.85–0.88)	0.87 (0.85–0.88)
Hypotonic uterine inertia	43,834 (9.50)	38,753 (8.89)	1.07 (1.05–1.08)	1.07 (1.05–1.08)
Hypertonic uterine inertia	512 (0.11)	401 (0.09)	1.21 (1.06–1.37)	1.21 (1.06–1.37)
Atonic hemorrhage	23,915 (5.18)	23,869 (5.47)	0.95 (0.93–0.96)	0.95 (0.93–0.96)
Blood transfusion	5032 (1.09)	4983 (1.14)	0.95 (0.92–0.99)	0.95 (0.92–0.99)
Instrumental delivery^b^	36,260 (11.17)	27,056 (8.79)	1.27 (1.25–1.29)	1.27 (1.26–1.29)
Cesarean delivery	136,728 (29.63)	128,039 (29.37)	1.01 (1.00–1.02)	1.01 (1.00–1.02)

			Mean difference (95% CI)	Adjusted mean difference^a^ (95% CI)
**Continuous variables (mean ± SD)**				
Gestational age at birth (weeks)	38.1 ± 2.4	38.3 ± 2.35	− 0.18 (− 0.19 to − 0.17)	− 0.18 (− 0.19 to − 0.17)
Birth weight (g)	2934 ± 546	2857 ± 522	77.6 (75.4 to 79.8)	77.3 (75.1 to 79.5)

All risk ratios and mean differences are calculated with females set as the reference.

*RR* risk ratio, *CI* confidence interval, *SD* standard deviation.

^a^Adjusted for maternal age, maternal height, maternal pre-pregnant body mass index, parity, and maternal smoking status during pregnancy.

^b^Analysis for instrumental delivery in 63,316 women (excludes those who received cesarean section).

We conducted a sensitivity analysis based on all 1,098,268 women with an imputed database including maternal age (n = 1280), parity (n = 3074), maternal height (n = 110,308), pre-pregnancy BMI (n = 163,510), conception method (n = 18,253), delivery mode (n = 9230), and fetal presentation at birth (n = 2). We conducted an analysis of delivery outcomes after excluding those with intrauterine fetal death (n = 1,091,228). The association between fetal gender and pregnancy complications and delivery outcomes was similar to that observed using the imputed data (Supplementary Tables S1 and S2 online).

## Discussion

Our study demonstrated that women carrying a male fetus had a significantly higher risk of preterm birth, abnormal uterine inertia, instrumental or cesarean delivery, and placental abruption. Those carrying a female fetus had a higher risk of preeclampsia, placenta accreta, a non-cephalic position at term, atonic hemorrhage, and maternal blood transfusion. Ours is the largest study showing an association between fetal gender and pregnancy outcomes as well as being the first study of Japanese women.

In line with previous studies^7–9,23^, we demonstrated that male fetal gender was associated with an increased risk of preterm delivery, very preterm delivery, and extremely preterm delivery. Although the etiology of this association remains unclear, several hypotheses have been proposed. One posits a difference in immune response according to fetal gender, with women carrying a male fetus being more likely to have chronic placental inflammation accompanied by incomplete remodeling of the spiral arterioles, leading to preterm birth^24–26^. This hypothesis is supported by a report of women carrying a male fetus having higher circulating levels of pro-inflammatory cytokines (e.g., granulocyte colony-stimulating factor, interleukin-21, and interleukin-33), which are responsible for excessive maternal inflammatory responses^27^. In an alternative hypothesis based on the findings of a study demonstrating a significant association between male fetal gender and increased CAM risk^28^, CAM contributed to an increased risk of preterm birth. However, our study failed to reveal a significant association between fetal gender and CAM, suggesting that another mechanism was responsible.

Studies of the association between fetal gender and preeclampsia are inconsistent. Some showed that the risk of preeclampsia was higher in women carrying a female fetus^29,30^, but others showed that the risk was higher in those carrying a male fetus^12,31^. While our finding of a higher risk of preeclampsia in women carrying a female fetus is reliable due to the large sample size (the largest study to date of this nature), further research is necessary to confirm the association.

Several mechanisms could explain why women carrying a female fetus had a higher preeclampsia risk. One possible explanation involves the gender-dependent differences in the renin–angiotensin system during early gestation. Reports have shown that among women carrying a female fetus, those with preeclampsia had a higher angiotensin level at early pregnancy than those without preeclampsia, while the angiotensin level was not elevated in women carrying a male fetus^32^. Another possible explanation is that the levels of human chorionic gonadotropin (hCG) vary by fetal gender. Several recent studies have reported that elevated hCG was a predictive marker of preeclampsia^33,34^ and that hCG levels were higher in women carrying a female fetus^35,36^. Although our study showed that women carrying a female fetus had a higher risk of preeclampsia, a previous study showed women carrying a male fetus have higher circulating levels of pro-inflammatory cytokines^27^, which are associated with an increased risk of preeclampsia and preterm birth. Thus, the underlying mechanisms may be complex, and future research focusing on pathophysiology is warranted.

We demonstrated that women carrying a male fetus had an increased risk of placental abruption, while those carrying a female fetus had an increased risk of placenta accreta. These findings correlate with the few studies that have examined the association between fetal gender and placental abruption or placenta accreta^37,38^. While several have demonstrated that women carrying a male fetus are likely to have higher circulating levels of pro-inflammatory cytokines, leading to the failure of spiral artery remodeling^4,26,27^, other studies have suggested that women carrying a male fetus also have a higher risk of placental abruption due to poor placental vascularization and deficient anchor in the matrix tissue^39,40^. In contrast, women carrying a male fetus were unlikely to have placenta accreta due to insufficient remodeling.

In line with previous findings^6,8–11,14,15^, we demonstrated that male fetal gender was an independent risk factor for instrumental and cesarean delivery. This may be explained by fetal gender-dependent differences in adaptation to distress during labor. Women carrying a male fetus are prone to having abnormal second-stage fetal heart rate patterns^41–43^; abnormal labor progress, including hyper- and hypotonic labor as previously demonstrated^44^; and macrosomia; which are all risk factors of instrumental and cesarean delivery^45,46^. Although the female fetus is likely to be in a non-cephalic position at birth, other factors, including abnormal fetal heart rate patterns, abnormal labor progress, and macrosomia, may be more influential contributors to an increased risk of cesarean section. In addition to the traditional complications of cesarean section, such as infection, surgical injury, and abnormal placentation in future pregnancies^47–49^, cesarean section could cause long-term risks in children, such as allergic disorders, childhood/adolescent obesity, autism spectrum disorders, and attention deficit hyperactivity disorder^50–52^. A higher rate of cesarean section among women carrying a male fetus could play some role in these associations.

Interestingly, we found that women carrying a female fetus had a significantly higher risk of atonic hemorrhage and maternal blood transfusion, despite having a lower risk of macrosomia, abnormal labor, and instrumental delivery. Studies have also demonstrated a higher risk of atonic hemorrhage and blood transfusion in women carrying a female fetus^15,53^. Further research is necessary to clarify the etiology of this confusing association.

The main strength of our study lies in the large sample size, enabling a reliable assessment of the association between fetal gender and various pregnancy complications and delivery outcomes, including the accurate assessment of rare outcomes, such as placenta accreta and blood transfusion, as well as small differences in fetal gender-related risks. However, our study has several limitations. First, because it was based on records of deliveries at mainly tertiary obstetric centers, our sample was likely to include higher risk pregnancies than the general population. Thus, our results, especially those of the absolute prevalence of each outcome by fetal gender, may not apply to the general population. Future replicative studies using a population-based database are warranted. Second, GDM, an important pregnancy outcome, was not analyzed due to the lack of data. Third, maternal endometriosis, which is associated with pregnancy outcomes^54^, was not recorded in our database. As our database was based on a collaboration of tertiary hospitals, a non-negligible number women may have endometriosis. Finally, our study was limited to analyzing the association between fetal gender and the risk of adverse pregnancy outcomes and, therefore, did not investigate the etiology of the association. Moreover, as the differences in the risk of most of the associations observed in this study were small, the results must be carefully interpreted. Nonetheless, our findings lay the groundwork for elucidating the etiology of the association between fetal gender and various pregnancy outcomes, even if their impact on clinical practice at present is small.

## Supplementary information

Supplementary Tables.
