# Supplementary material for: Differences in pregnancy complications and outcomes by fetal gender among Japanese women: a multicenter cross-sectional study
Source: Sci Rep. 2020 Nov 2;10:18810. doi: 10.1038/s41598-020-75969-8 (PMC7606487; doi:10.1038/s41598-020-75969-8)
Supplement: Supplementary file 1 — Supplementary Tables. [file 41598_2020_75969_MOESM1_ESM.pdf]

Differences in pregnancy complications and outcomes by fetal gender among Japanese women: a multicenter cross-sectional study

Satoru Funaki, Kohei Ogawa, Nobuaki Ozawa, Aikou Okamoto, Naho Morisaki & Haruhiko Sago

Supplemental Table S1. Association between fetal sex and pregnancy complications based on imputed dataset. (n=1,0,98,268)

**Outcomes**		**Crude RR**	**Adjusted RR[a]**
Preeclampsia	Male	0.92 (0.89–0.94)	0.91 (0.89–0.94)
	Female	Reference	Reference
HELLP syndrome	Male	1.06 (0.95–1.18)	1.06 (0.95–1.17)
	Female	Reference	Reference
Placental abruption	Male	1.14 (1.10–1.19)	1.14 (1.10–1.19)
	Female	Reference	Reference
Placenta accreta	Male	0.90 (0.85–0.95)	0.90 (0.85–0.95)
	Female	Reference	Reference
Placenta previa	Male	1.01 (0.98–1.04)	1.01 (0.98–1.04)
	Female	Reference	Reference
Intrauterine fetal death	Male	1.01 (0.96–1.06)	1.01 (0.96–1.05)
	Female	Reference	Reference
Chorioamnionitis	Male	1.02 (0.98–1.07)	1.02 (0.98–1.06)
	Female	Reference	Reference
Cord prolapse	Male	1.23 (0.98–1.54)	1.23 (0.96–1.58)
	Female	Reference	Reference
Amniotic fluid embolism	Male	0.96 (0.69–1.34)	0.96 (0.69–1.34)
	Female	Reference	Reference

RR: Risk ratio, HELLP: preeclampsia, hemolysis, elevated liver enzymes, and low platelet count. [a]Adjusted for maternal age, maternal height, maternal pre-pregnant body mass index, parity, conception method, and maternal smoking status during pregnancy.

Supplemental Table S2. Association between fetal sex and delivery outcomes among women with live birth based on imputed dataset. (n=1,091,228)

Outcomes		Crude RR	Adjusted RR[a]
**Categorized variables**			
Preterm delivery (<37 weeks)	Male	1.19 (1.18–1.21)	1.19 (1.18–1.21)
	Female	Reference	Reference
Very preterm delivery (<32 weeks)	Male	1.10 (1.08–1.13)	1.10 (1.08–1.13)
	Female	Reference	Reference
Extremely preterm delivery (<28 weeks)	Male	1.08 (1.03–1.12)	1.08 (1.03–1.12)
	Female	Reference	Reference
Macrosomia	Male	1.82 (1.74–1.90)	1.81 (1.73–1.89)
	Female	Reference	Reference
Low birth weight	Male	0.87 (0.86–0.87)	0.87 (0.86–0.87)
	Female	Reference	Reference
Non-cephalic position at birth	Male	0.87 (0.85–0.88)	0.87 (0.85–0.88)
	Female	Reference	Reference
Hypotonic uterine inertia	Male	1.07 (1.06–1.08)	1.07 (1.06–1.09)
	Female	Reference	Reference
Hypertonic uterine inertia	Male	1.26 (1.11–1.42)	1.26 (1.12–1.42)
	Female	Reference	Reference
Atonic hemorrhage	Male	0.94 (0.93–0.96)	0.94 (0.93–0.96)
	Female	Reference	Reference
Blood transfusion	Male	0.95 (0.92–0.99)	0.96 (0.92–0.99)
	Female	Reference	Reference
Instrumental delivery	Male	1.27 (1.25–1.29)	1.27 (1.26–1.29)
	Female	Reference	Reference
Cesarean delivery	Male	1.01 (1.00–1.02)	1.01 (1.00–1.01)
	Female	Reference	Reference
**Continuous variables**		**Mean difference (95% CI)**	**Adjusted mean difference[a] (95% CI)**
Gestational age at birth (weeks)		−0.18 (−0.19 to −0.17)	−0.18 (−0.19 to −0.17)
Birth weight (g)		78.2 (76.2 to 80.2)	78.1 (76.0 to 80.3)

All mean differences are calculated using females as the reference. RR: risk ratio, CI: confidence interval.

[a]Adjusted for maternal age, maternal height, maternal pre-pregnant body mass index, parity, and maternal smoking status during pregnancy.
